# Networks for Nonlinear Diffusion Problems in Imaging

**DOI:** 10.1007/s10851-019-00901-3

**Published:** 2019-09-13

**Authors:** S. Arridge, A. Hauptmann

**Affiliations:** 1 Department of Computer Science, University College London, London, UK; 2 Research Unit of Mathematical Sciences, University of Oulu, Oulu, Finland

**Keywords:** Neural networks, Deep learning, Partial differential equations, Nonlinear diffusion, Image flow, Nonlinear inverse problems

## Abstract

A multitude of imaging and vision tasks have seen recently a major transformation by deep learning methods and in particular by the application of convolutional neural networks. These methods achieve impressive results, even for applications where it is not apparent that convolutions are suited to capture the underlying physics. In this work, we develop a network architecture based on nonlinear diffusion processes, named *DiffNet*. By design, we obtain a nonlinear network architecture that is well suited for diffusion-related problems in imaging. Furthermore, the performed updates are explicit, by which we obtain better interpretability and generalisability compared to classical convolutional neural network architectures. The performance of DiffNet is tested on the inverse problem of nonlinear diffusion with the Perona–Malik filter on the STL-10 image dataset. We obtain competitive results to the established U-Net architecture, with a fraction of parameters and necessary training data.

## Introduction

We are currently undergoing a paradigm shift in imaging and vision tasks from classical analytic to learning and data-based methods. In particular, this shift is driven by deep learning and the application of convolutional neural networks (CNN). Whereas highly superior results are obtained, interpretability and analysis of the involved processes is a challenging and ongoing task [20, 24].

In a general setting, deep learning proposes to develop a nonlinear mapping $A_{\varTheta } : X\rightarrow Y$ between elements of two spaces *X*, *Y* (which may be the same) parametrised by a finite set of parameters $\varTheta$, which need to be learned:

1.1 $$\begin{aligned} g = A_{\varTheta } f \end{aligned}$$

This learning-based approach is in marked contrast to classical methods with a physical interpretation of the process (1.1) for both computational modelling of data given a physical model (which we call a *forward problem*) and the estimation of parameters of a physical model from measured, usually noisy, data (which we call an *inverse problem*). Several recent papers have discussed the application of deep learning in both forward [23, 31, 36, 37, 40, 41] and inverse [21, 22, 29, 42–44] problems. Several questions become essential when applying learned models in such cases including:

- how and to what extent can learned models replace physical models?
- how do learned models depend on training protocols and how well do they generalise?
- what are appropriate architectures for the learned models, what is the size of the parameter set $\varTheta$ that needs to be learned, and how can these be interpreted?

In this paper, we aim to answer some of these questions, by taking a few steps back and looking at an analytic motivation for network architectures. Here, we consider in particular mappings between images *u* in dimension *d*, i.e. $X = Y = L^p(\varOmega \subset \mathbb {R}^d)$, for which there exist several widely used linear and nonlinear mappings *A* defined by differential and/or integral operators. For example, a general linear integral transform such as

1.2 $$\begin{aligned} u^\mathrm{obs}(x) = (A u^\mathrm{true})(x) = \int _{\varOmega } K(x,y) u^\mathrm{true}(y) {\mathrm{d}}y \end{aligned}$$

includes stationary convolution as a special case if the kernel is translation invariant, i.e. $K(x,y) \equiv K(x-y)$. If the kernel depends on the image, i.e. $K(x,y) \equiv K(x,y; u)$, then (1.2) becomes nonlinear. Alternatively, the forward mapping may be modelled as the end-point of an evolution process which becomes nonlinear if the kernel depends on the image state $K(x,y,t) \equiv K(x,y;u(t))$; see Eq. (2.7) below for a specific example.

Furthermore, a widely studied class of image mappings is characterised by defining the evolution through a partial differential equation (PDE) where flow is generated by the local structure of *u* [25, 34, 38], such that

1.3 $$\begin{aligned} u_t = F\left( u,\nabla u, \frac{\partial ^{2} {u}}{\partial {x_i} \partial {x_j}},\ldots \right) \, . \end{aligned}$$

These problems in general do not admit an explicit integral transform representation and are solved instead by numerical techniques, but since they depend on a physical model, described by the underlying PDE, they can be analysed and understood thoroughly [39]. This also includes a statistical interpretation [19] as hyperpriors in a Bayesian setting [5]. Furthermore, models like (1.3) can be used to develop priors for nonlinear inverse problems, such as optical tomography [8, 15] and electrical impedance tomography [13].

Motivated by the success of these analytic methods to imaging problems in the past, we propose to combine physical models with data-driven methods to formulate network architectures for solving both forward and inverse problems that take the underlying physics into account. We limit ourselves to the case where the physical model is of diffusion type, although more general models could be considered in the future. The leading incentive is given by the observation that the underlying processes in a neural network do not need to be limited to convolutions.

Similar ideas of combining partial differential equations with deep learning have been considered earlier. For instance, this is done by learning of a PDE via optimal control [26], as well as deriving CNN architectures motivated by diffusion processes [6], deriving stable architectures by drawing connections to ordinary differential equations [11] and constraining CNNs [33] by the interpretation as a partial differential equation. ‘PDE-NET 2.0’ [27, 28] is a recent example of a network architecture designed to learn dynamic PDEs assumed to be of the form (1.3) where the function *F* is learned as a polynomial in convolution filters with appropriate vanishing moments. Another interpretation of our approach can be seen as introducing the imaging model into the network architecture; such approaches have led to a major improvement in reconstruction quality for tomographic problems [2, 14, 17].

This paper is structured as follows. In Sect. 2, we review some theoretical aspects of diffusion processes for imaging and the inversion based on theory of partial differential equations and differential operators. We formulate the underlying conjecture for our network architecture that the diffusion process can be inverted by a set of local non-stationary filters. In the following, we introduce the notion of *continuum networks* in Sect. 3 and formally define the underlying *layer operator* needed to formulate network architectures in a continuum setting. We draw connections to the established convolutional neural networks in our continuum setting. We then proceed to define the proposed layer operators for diffusion networks in Sect. 4 and derive an implementable architecture by discretising the involved differential operator. In particular, we derive a network architecture that is capable of reproducing inverse filtering with regularisation for the inversion of nonlinear diffusion processes. We examine the reconstruction quality of the proposed *DiffNet* in following Sect. 5 for an illustrative example of deconvolution and the challenging inverse problem of inverting nonlinear diffusion with the Perona–Malik filter. We achieve results that are competitive to popular CNN architectures with a fraction of the amount of parameters and training data. Furthermore, all computed components that are involved in the update process are interpretable and can be analysed empirically. In Sect. 6, we examine the generalisablity of the proposed network with respect to necessary training data. Additionally, we empirically analyse the obtained filters and test our underlying conjecture. Section 7 presents some conclusions and further ideas.

## Diffusion and Flow Processes for Imaging

In the following, we want to explore the possibility to include a nonlinear process as an underlying model for network architectures. Specifically, the motivation for this study is given by diffusion processes that have been widely used in imaging and vision. Let us consider here the general diffusion process in $\mathbb {R}^d$, then on a fixed time interval with some diffusivity $\gamma$, to be defined later, we have

2.1 $$\begin{aligned} \left\{ \begin{array}{lll} \partial _t u\ &{}=\ \nabla \cdot (\gamma \nabla u) &{} \text {in }\mathbb {R}^d\times (0,T] \\ u(x,0)\ &{}=\ u_0(x) &{} \text {in } \mathbb {R}^d. \end{array} \right. \end{aligned}$$

### Remark 1

When considering bounded domains $\varOmega \subset \mathbb {R}^d$, we will augment (2.1) with boundary conditions on $\partial \varOmega$. We return to this point in Sect. 4.

In the following, we denote the spatial derivative by

2.2 $$\begin{aligned} \left( \mathcal {L}(\gamma )u\right) (x,t):= \nabla \cdot (\gamma \nabla u(x,t)). \end{aligned}$$

Let us first consider the *isotropic diffusion* case, then the differential operator becomes the spatial Laplacian $\mathcal {L}(\gamma = 1)= \Delta$. In this case, the solution of (2.1) at time *T* is given by convolution with a *Green’s function*

2.3 $$\begin{aligned} u_T(x) = G_{\sqrt{2T}}(x)*u_0(x) \end{aligned}$$

where $G_{\sqrt{2T}} = \frac{1}{(4\pi T)^{d/2}}\exp \left[ -\frac{x^2}{4T}\right]$ in dimension *d* and we recall that the convolution of two functions $f,g\in L^1(\mathbb {R}^d)$ is defined by

2.4 $$\begin{aligned} (g*f)(x) = \int _{\mathbb {R}^d} g(x-y)f(y) {\mathrm{d}}y. \end{aligned}$$

### Definition 1

The *Green’s operator* is defined with the Green’s function as its kernel

2.5 $$\begin{aligned} \mathcal {G}_{\frac{1}{2}\sigma ^2} u := \int _{\mathbb {R}^d} \frac{u(x)}{(2\pi \sigma ^2)^{d/2}}\exp \left[ -\frac{|x-y|^2}{2\sigma ^2}\right] {\mathrm{d}}y \end{aligned}$$

by which we have

$$\begin{aligned} u_T(x) = \mathcal {G}_\mathrm{T} u_0 (x) \end{aligned}$$

In the general case for an *anisotropic diffusion flow* (ADF), we are interested in a scalar *diffusivity*$\gamma \in [0,1]$ that depends on *u* itself, i.e.

2.6 $$\begin{aligned} \partial _t u= \nabla \cdot \left( \gamma (u) \nabla u\right) \end{aligned}$$

This is now an example of a nonlinear evolution

2.7 $$\begin{aligned} u_T(x) = \mathcal {K}_T u_0 = \int _0^T\int _{\mathbb {R}^d} K^\mathrm{ADF}(x,y,u(y,t) ) u_0(y) {\mathrm{d}}y {\mathrm{d}}t \nonumber \\ \end{aligned}$$

where $K^\mathrm{ADF}(x,y,u(x,t))$ is now a non-stationary, nonlinear and time-dependent kernel. In general, there is no explicit expression for $K^\mathrm{ADF}$ and numerical methods are required for the solution of (2.6).

### Remark 2

Not considered here, but a possible extension to (2.6) is where $\gamma$ is a tensor, which for $d=2$ takes the form

$$\begin{aligned} \partial _t u = \nabla \cdot \begin{pmatrix}\gamma _{11} &{} \gamma _{12} \\ \gamma _{12} &{} \gamma _{22} \end{pmatrix} \nabla u \end{aligned}$$

Furthermore, extensions exist for the case where *u* is vector or tensor valued. We do not consider these cases here, see [38] for an overview.

### Forward Solvers

First of all, let us establish a process between two states of the function *u*. Integrating over time from $t=t_0$ to $t = t_1=t_0+\delta t$ yields

$$\begin{aligned} \int _{t_0}^{t_1} \partial _t u(x,t) \mathrm {d}t= \int _{t_0}^{t_1} \left( \mathcal {L}(\gamma )u\right) (x,t) \mathrm {d}t. \end{aligned}$$

Note that the left-hand side can be expressed as $\int _{t_0}^{t_1} \partial _t u(x,t) \mathrm {d}t= u(x,t_1)-u(x,t_0)$ and we denote the right-hand side by an integral operator $\mathcal {A}_{\delta t}(\gamma )$, such that

2.8 $$\begin{aligned} (\mathcal {A}_{\delta t}(\gamma )u)(x,t_0) := \int _{t_0}^{t_1=t_0+\delta t} \left( \mathcal {L}(\gamma )u\right) (x,t) \mathrm {d}t. \end{aligned}$$

In the following, we denote the solution of (2.1) at time instances $t_n$ as $u^{(n)}=u(x,t_n)$. Then, we can establish a relation between two time instances of *u* by

2.9 $$\begin{aligned} u^{(n+1)} = ({\hbox {Id}} + \mathcal {A}_{\delta t}(\gamma ) )u^{(n)}= u^{(n)} + \int _{t_n}^{t_{n+1}} \mathcal {L}(\gamma )u(x,t) \mathrm {d}t,\nonumber \\ \end{aligned}$$

where Id denotes the identity and $t_{n+1}=t_n+\delta t$.

Since we cannot compute $u^{(n+1)}$ by (2.9) without the explicit knowledge of the (possibly time dependent) diffusivity $\gamma$, it is helpful to rather consider a fixed diffusivity at each time instance $\gamma ^{(n)}=\gamma (x,t=t_n)$, or $\gamma ^{(n)}=\gamma (u(x,t=t_n))$ in the nonlinear case; then, by using the differential operator (2.2), we have an approximation of (2.8) by

$$\begin{aligned} \delta t \mathcal {L}(\gamma ^{(n)}) u^{(n)}= \delta t (\nabla \cdot \gamma ^{(n)} \nabla u^{(n)}) \approx \mathcal {A}_{\delta t}(\gamma ) u^{(n)}. \end{aligned}$$

We can now solve (2.6) approximately by iterating for time steps $\delta t$ using either an *explicit* scheme

2.10 $$\begin{aligned} \mathcal {D}_{\delta t}^{\mathrm{Expl}}(\gamma ^{(n)})u^{(n)} = \left( {\hbox {Id}} + \delta t {\mathcal {L}}(\gamma ^{(n)}) \right) u^{(n)}\,, \end{aligned}$$

or an *implicit* scheme

2.11 $$\begin{aligned} \mathcal {D}_{\delta t}^{\mathrm{Impl}}(\gamma ^{(n)})u^{(n)} = \left( {\hbox {Id}} - \delta t {\mathcal {L}}(\gamma ^{(n)}) \right) ^{-1} u^{(n)}\,, \end{aligned}$$

whereas (2.10) is stable only if CFL conditions are satisfied and (2.11) is unconditionally stable, they are both only accurate for sufficiently small steps $\delta t$. In fact, by the Neumann series, the schemes are equivalent for small $\delta t$ as

2.12 $$\begin{aligned} \left( {\hbox {Id}} - \delta t {\mathcal {L}}(\gamma ) \right) ^{-1} = {\hbox {Id}} + \delta t {\mathcal {L}}(\gamma ) + \mathcal {O}((\delta t)^2)\, \end{aligned}$$

and coincide with the integral formulation of (2.9).

It is also useful to look at the Green’s function’s solutions.

#### Lemma 1

Consider the isotropic case with $\gamma \equiv 1$. Then, we may write, with time steps $\delta t = T/N$,

2.13 $$\begin{aligned} \begin{aligned} \mathcal {G}_Tu_0&= G_{\sqrt{2 T}} *u_0 \\&= \underbrace{G_{\sqrt{2 \delta t}} *\ldots *G_{\sqrt{2 \delta t}}}_{N -times } *u_0 = \underbrace{\mathcal {G}_{\delta t}\circ \ldots \mathcal {G}_{\delta t}}_{N -times } \circ u_0 \end{aligned}\nonumber \\ \end{aligned}$$

#### Proof

Take the Fourier Transform^1^

$$\begin{aligned} \hat{G}_{\sigma }(k) = {\mathcal {F}}_{x\rightarrow k} G_{\sigma }(x) = \mathrm{e}^{-\frac{\sigma ^2 k^2}{2}} \end{aligned}$$

and use the convolution theorem to give

$$\begin{aligned} \hat{G}_{\sqrt{2 T}}(k) \hat{u}_0(k)= & {} \left( \varPi _{n=1}^N\hat{G}_{\sqrt{2 \delta t}}(k) \right) \hat{u}_0(k) \\ \mathrm{e}^{-{k^2T} } \hat{u}_0(k)= & {} \left( \mathrm{e}^{-{k^2 \delta t} } \right) ^N \hat{u}_0(k) = \mathrm{e}^{-{k^2 N\delta t} } \hat{u}_0(k), \end{aligned}$$

which gives the claim. $\square$

Let us also note that in Fourier domain, by Taylor series expansion, we have

$$\begin{aligned} \exp (-k^2 \delta t) \rightarrow 1 - k^2\delta t + \frac{1}{2} k^4 (\delta t)^2 - \cdots \,, \end{aligned}$$

and therefore, in the spatial domain, the finite difference step and the Gaussian convolution step are the same

$$\begin{aligned} \begin{aligned} \lim _{\delta t \rightarrow 0}\left( G_{\sqrt{2 \delta t} }*u_0 \right)&= \left( {\hbox {Id}} + \delta t \Delta \right) *u_0 \\&= \lim _{\delta t \rightarrow 0}\left( {\hbox {Id}} - \delta t \Delta \right) ^{-1}u_0 \,. \end{aligned} \end{aligned}$$

### Inverse Filtering

Let us now consider the inverse problem of reversing the diffusion process. That is we have $u_T$ and aim to recover the initial condition $u_0$. This is a typical ill-posed problem as we discuss in the following.

#### Isotropic Case $\gamma \equiv 1$

As the forward problem is represented as convolution in the spatial domain, the inverse mapping $u_T \mapsto u_0$ is a (stationary) *deconvolution*. We remind that $\hat{u}_T=\mathrm{e}^{-{k^2 T} } \hat{u}_0(k)$; then, the inversion is formally given by division in the Fourier domain as

2.14 $$\begin{aligned} u_0(x) = {\mathcal {F}}^{-1}_{k\rightarrow x}\left[ \hat{u}_T(k) \mathrm{e}^{k^2 T} \right] \,. \end{aligned}$$

However, we note:

(i) The factor $\mathrm{e}^{k^2 T}$ is unbounded, and hence, the equivalent convolution kernel in the spatial domain does not exist.
(ii) Equation (2.14) is unstable in the presence of even a small amount of additive noise, and hence, it has to be *regularised* in practice.

Nevertheless, let us consider formally with $\mathrm{e}^{k^2 T} = \left( \mathrm{e}^{k^2 \delta t} \right) ^N$ that by Taylor series, we get

$$\begin{aligned} \begin{aligned} {\mathcal {F}}^{-1}_{k\rightarrow x}\mathrm{e}^{k^2 \delta t}&\approx {\mathcal {F}}^{-1}_{k\rightarrow x}\left[ 1 + k^2\delta t + \frac{1}{2}(k^2\delta t)^2 + \cdots \right] \\&= 1 - \delta t \Delta + \mathcal {O}((\delta t)^2)\,. \end{aligned} \end{aligned}$$

Motivated by this, we define an operator for the inversion process

2.15 $$\begin{aligned} \mathcal {E}^\mathrm{iso}_{\delta t} u := \left( {\hbox {Id}} - \delta t \Delta \right) u \, \simeq \mathcal {G}^{-1}_{\delta t} u. \end{aligned}$$

Clearly, $\mathcal {E}_{\delta t}^\mathrm{iso}$ coincides with the inverse of the implicit update in (2.11), and

2.16 $$\begin{aligned} \tilde{u}_{0} = \underbrace{{\mathcal {E}}^\mathrm{iso}_{\delta t} \circ \ldots \circ {\mathcal {E}}^\mathrm{iso}_{ \delta t}}_{N -times } \circ \, u_T \end{aligned}$$

is an estimate for the deconvolution problem which (in the absence of noise) is correct in the limit

2.17 $$\begin{aligned} \lim _{\delta t \rightarrow 0} \tilde{u}_{0} \rightarrow u_0. \end{aligned}$$

#### Anisotropic Case

In this case, the diffused function is given by (2.7). Following Lemma 1, we may put

2.18 $$\begin{aligned} u_T = \mathcal {K}_T u_0 \simeq \tilde{u}_T := \mathcal {D}_{\delta t}^{\mathrm{Expl}}(\gamma ^{(N-1)}) \circ \cdots \circ \mathcal {D}_{\delta t}^{\mathrm{Expl}}(\gamma ^{(0)}) u_0 \nonumber \\ \end{aligned}$$

and we also have

2.19 $$\begin{aligned} \lim _{\delta t \rightarrow 0}\tilde{u}_{T} \rightarrow u_T. \end{aligned}$$

##### Conjecture 1

There exists a set of local (non-stationary) filters $\mathcal {E}_{\delta t} (\zeta )$ where

2.20 $$\begin{aligned} \mathcal {E}_{\delta t} (\zeta ) u = u - \delta t\int _{{\mathbb {R}}^d} \zeta (x,y) u(y) {\mathrm{d}}y \end{aligned}$$

and where $\zeta (x,y)$ has only local support and such that

2.21 $$\begin{aligned} u_0 = \mathcal {K}_T^{-1}u_T \quad \simeq \quad \tilde{u}_0:= \mathcal {E}_{\delta t} ( \zeta ^{(N-1)})\circ \cdots \circ \mathcal {E}_{\delta t} ( \zeta ^{(0)}) u_T \,. \nonumber \\ \end{aligned}$$

##### Remark 3

(Unsharp Masking) We recall that a simple method for “deconvolution” is called *Unsharp Masking* which is usually considered as

$$\begin{aligned} u^\mathrm{obs} \mapsto \tilde{u} = u + \epsilon (u^\mathrm{obs}-G_{\sigma }*u^\mathrm{obs} ) \end{aligned}$$

for some blur value $\sigma$ and sufficiently small $\epsilon$. By similar methods as above, we find

$$\begin{aligned} \hat{\tilde{u} }(k)= & {} \hat{u}^\mathrm{obs}(k) +\epsilon \left( {\hbox {Id}} - \mathrm{e}^{-\frac{\sigma ^2k^2}{2}} \right) \hat{u}^\mathrm{obs}(k) \\\simeq & {} \left( {\hbox {Id}} + {\frac{\epsilon \sigma ^2k^2}{2}} \right) \hat{u}^\mathrm{obs}(k)\\ \Rightarrow \tilde{u}\simeq & {} \left( {\hbox {Id}} - \frac{\epsilon \sigma ^2}{2} \Delta \right) u^\mathrm{obs}(x)\, . \end{aligned}$$

We may choose to interpret the operators ${\mathcal {E}}_{\delta t}(\zeta )$ as a kind of “non-stationary unsharp masking”.

For the presented problem of *non-stationary nonlinear* blind deconvolution/inverse filtering, we are not aware of any suitable classical methods. For a recent study that discusses backward diffusion, see [4].

### Discretisation

We introduce the definition of a sparse matrix operator representing local non-stationary convolution

#### Definition 2

$\mathsf {W}$ is called a *Sparse Sub-Diagonal* (SSD) matrix if its nonzero entries are all on sub-diagonals corresponding to the local neighbourhood of pixels on its diagonal.

Furthermore, we are going to consider that a class of SSD matrices $\mathsf {W}(\zeta )$ with learned parameters $\zeta$ can be decomposed as $\mathsf {W}(\zeta ) = \mathsf {S}(\zeta ) + \mathsf {L}(\zeta )$ where $\mathsf {S}$ is *smoothing* and $\mathsf {L}(\zeta )$ is *zero-mean*, i.e. $\mathsf {L}(\zeta )$ has one zero eigenvalue such that its application to a constant image gives a zero valued image

$$\begin{aligned} \mathsf {L}(\zeta ) \mathbb {1} = 0 \end{aligned}$$

In the following, we restrict ourselves to the typical 4-connected neighbourhood of pixels in dimension $d=2$. For the numerical implementation, we have the Laplacian stencil

$$\begin{aligned} \Delta \quad \rightarrow \quad \mathsf {L}_{\Delta } = \begin{pmatrix} &{}1&{}\\ 1&{}-4&{}1\\ &{}1&{} \end{pmatrix} \end{aligned}$$

from which we have that $\mathsf {L}_{\Delta }$ is zero mean. Similarly, we will have for the numerical approximation of ${\mathcal {E}}^\mathrm{iso}_{\delta t}$ the matrix operator

$$\begin{aligned} {\hbox {Id}} - \delta t \Delta \quad \rightarrow \quad {\mathsf {E}}^\mathrm{iso}_{\delta t} = \begin{pmatrix} &{} 0 &{}\\ 0 &{} 1 &{} 0 \\ &{} 0 &{} \end{pmatrix} - \begin{pmatrix} &{}\delta t&{}\\ \delta t&{}-4\delta t&{}\delta t\\ &{}\delta t&{} \end{pmatrix}. \end{aligned}$$

Further we conjecture that in the numerical setting, $\mathcal {E}_{\delta t} (\zeta )$ is approximated by the sum of identity plus a SSD matrix operator as

2.22 $$\begin{aligned} \begin{aligned} \mathcal {E}_{\delta t} (\zeta ) \,&\sim \, {{\hbox {Id}}} - \delta t \mathcal {L}(\zeta ) \rightarrow \mathsf {Id} - \mathsf {L}_{\delta t}(\zeta ) \\&\sim \, \mathsf {E}_{\delta t} (\zeta ) = \begin{pmatrix} &{} 0 &{}\\ 0 &{} 1 &{} 0 \\ &{} 0 &{} \end{pmatrix} - \delta t \begin{pmatrix} &{}\zeta _1 &{}\\ \zeta _2&{}-\sum _i \zeta _i &{}\zeta _4 \\ &{} \zeta _3 &{} \end{pmatrix}. \end{aligned} \end{aligned}$$

In the presence of noise, the inverse operator $\mathcal {E}_{\delta t} (\zeta )$ can be explicitly regularised by addition of a smoothing operation

2.23 $$\begin{aligned} \tilde{\mathcal {E}}_{\delta t} (\zeta ) = \mathcal {E}_{\delta t} (\zeta ) +\alpha \mathcal {S} \rightarrow \mathsf {Id} - \mathsf {L}_{\delta t}(\zeta ) + \alpha \mathsf {S} =: \mathsf {Id} - \mathsf {W}_{\delta t}(\zeta )\nonumber \\ \end{aligned}$$

whereas in classical approaches to inverse filtering, the regularisation operator would be defined *a priori*, the approach in this paper is to learn the operator $\mathsf {W}$ and interpret it as the sum of a differentiating operator $\mathsf {L}$ and a (learned) regulariser $\mathsf {S}$. This is discussed further in Sect. 4

## Continuum Networks

Motivated by the previous section, we aim to build network architectures based on diffusion processes. We first discuss the notion of (neural) networks in a continuum setting for which we introduce the concept of a *continuum network* as a mapping between function spaces. That is, given a function on a bounded domain $\varOmega \subset \mathbb {R}^d$ with $f\in L^p(\varOmega )$, we are interested in finding a nonlinear parametrised operator $\mathcal {H}_\varTheta :L^p(\varOmega )\rightarrow L^p(\varOmega )$ acting on the function *f*. We will consider in the following the case $p\in \{1,2\}$; extensions to other spaces depend on the involved operations and will be the subject of future studies.

We will proceed by defining the essential building blocks of a continuum network and thence to discuss specific choices to obtain a continuum version of the most common convolutional neural networks. Based on this, we will then introduce our proposed architecture as a diffusion network in the next chapter.

### Formulating a Continuum Network

The essential building blocks of a deep neural network are obviously the several layers of neurons, but since these have a specific notion in classical neural networks, see, for instance, [35], we will not use the term of neurons to avoid confusion. We rather introduce the concept of layers and channels as the building blocks of a *continuum network*. In this construction, each layer consists of a set of functions on a product space and each function represents a channel.

#### Definition 3

(*Layer and channels*) For $k\in \mathbb {N}_0$, let $F_k=\{f^k_1,f^k_2,\cdots , f^k_I\}$ be a set of functions $f^k_i\in L^p(\varOmega )$ for $i\in \mathbb {I}=\{1,\dots ,I\}$, $I\ge 1$. Then, we call: $F_k$ the layer *k* with *I* channels and corresponding index set $\mathbb {I}$.

The continuum network is then built by defining a relation or operation between layers. In the most general sense, we define the concept of a layer operator for this task.

#### Definition 4

(*Layer operator*) Given two layers $F_k$ and $F_{t}$, $k\ne t$, with channel index set $\mathbb {I},\ \mathbb {J}$, respectively, we call the mapping $\mathcal {H}:\bigotimes _\mathbb {I}L^p(\varOmega ) \rightarrow \bigotimes _\mathbb {J}L^p(\varOmega )$ with

$$\begin{aligned} \mathcal {H}F_{k} = F_t \end{aligned}$$

a layer operator. If the layer operator depends on a set of parameters $\varTheta$, then we write $\mathcal {H}_{\varTheta }$.

We note that for simplicity, we will not index the set of parameters, i.e. $\varTheta$ generally stands for both all involved parameters of each layer separately, or the whole network. The classical structure for layer operators follows the principle of affine linear transformations followed by a nonlinear operation. Ideally, the affine linear transformation should be parameterisable by a few parameters, whereas the nonlinear operation is often fixed and acts pointwise. A popular choice is the maximum operator also called the “Rectified Linear Unit”:

$$\begin{aligned} \mathrm {ReLU}:L^p(\varOmega )\rightarrow L^p(\varOmega ), f\mapsto \max (f,0). \end{aligned}$$

The continuum network is then given by the composition of all involved layer functions. For example, in monochromatic imaging applications, we typically have an input image $f_0$ and a desired output $f_K$ with several layer functions in-between that perform a specific task such as denoising or sharpening. In this case, the input and output consist of one channel, i.e. $|F_0|=|F_K|=1$; consequently, for colour images (in RGB), we have $|F_0|=|F_K|=3$.

### Continuum Convolutional Networks

Let us now proceed to discuss a specific choice for the layer operator, namely convolutions. With this choice, we will obtain a continuum version of the widely used convolutional neural networks, which we will call here a *continuum convolutional network*, to avoid confusion with the established convolutional neural networks (CNN). We note that similar ideas have been addressed as well in [2].

Let us further consider linearly ordered network architectures that means each layer operator maps between consecutive layers. The essential layer operator for a continuum convolutional network is then given by the following definition.

#### Definition 5

(*Convolutional layer operator*) Given two layers $F_{k-1}$ and $F_{k}$ with channel index set $\mathbb {I},\ \mathbb {J}$, respectively, let $b_j\in \mathbb {R}$ and $\omega _{i,j}\in L^p(\varOmega )$, with compact support in $\varOmega$, be the layer operator’s parameters for all $i\in \mathbb {I},\ j\in \mathbb {J}$. We call $\mathcal {C}^{(k)}_{\varTheta ,\varphi }$ the convolutional layer operator for layer *k*, if for each output channel

3.1 $$\begin{aligned} \mathcal {C}^{(k)}_{\varTheta ,\varphi }F_{k-1}=\varphi \left[ b_j + \sum _{i\in \mathcal {I}} \omega _{i,j} *f^{k-1}_{i} \right] =f^k_{j}, \ j \in \mathbb {J},\nonumber \\ \end{aligned}$$

with a pointwise nonlinear operator $\varphi : L^p(\varOmega )\rightarrow L^p(\varOmega )$.

If the layer operator does not include a nonlinearity, we write $\mathcal {C}_{\varTheta ,{\mathrm{Id}}}$. Now, we can introduce the simplest convolutional network architecture by applying $K\ge 1$ convolutional layer operators consecutively.

#### Definition 6

(*K-layer Continuum Convolutional Network*) Let $K\ge 1$, then we call the composition of *K* convolutional layer operator, denoted by $\mathcal {C}^K_\varTheta$, a K-layer continuum convolutional network, such that

3.2 $$\begin{aligned} \mathcal {C}^K_{\varTheta ,\varphi } = {\mathcal {C}^{(K)}_{\varTheta ,\varphi } \circ \dots \circ \mathcal {C}^{(1)}_{\varTheta ,\varphi }}, \mathcal {C}^K_\varTheta F_0=F_K. `\end{aligned}$$

In the following, we will also refer to a *K*-layer CNN as the practical implementation of a *K*-layer continuum convolutional network. A popular network architecture that extends this simple idea is given by a residual network (ResNet) [18], that is, based on the repetition of a 2-layer CNN with a residual connection, that consists of addition. That is, the network learns a series of additive updates to the input. The underlying structure in ResNet is the repeated application of the following *residual block* given by

3.3 $$\begin{aligned} \mathcal {R}_{\varTheta ,\varphi } = \mathcal {C}^{(2)}_{\varTheta ,{\mathrm{Id}}}\circ \mathcal {C}^{(1)}_{\varTheta ,\varphi } + {\hbox {Id}}, \mathcal {R}_{\varTheta ,\varphi }F_0=F_2. \end{aligned}$$

Note that the second convolutional layer does not include a nonlinearity. Furthermore, it is necessary that $|F_0|=|F_2|$, but typically, it is often chosen such that $|F_0|=|F_1|=|F_2|$. The full continuum ResNet architecture can then be summarised as follows. Let $K\ge 1$, then the composition of *K* residual blocks, denoted by $\mathcal {R}^K_{\varTheta ,\varphi }$, defines a *K*-block continuum ResNet

3.4 $$\begin{aligned} \mathcal {R}^K_{\varTheta ,\varphi } = \mathcal {C}^{(K+1)}_{\varTheta ,\varphi } \circ \mathcal {R}^{(K)}_{\varTheta ,\varphi } \circ \dots \circ \mathcal {R}^{(1)}_{\varTheta ,\varphi }\circ \mathcal {C}^{(0)}_{\varTheta ,\varphi }. \end{aligned}$$

Note that the two additional convolutional layers in the beginning and end are necessary to raise the cardinality of the input/output layer to the cardinality needed in the residual blocks. A complete K-block ResNet then consists of $2(K+1)$ layers. Note that in the original work [18], the network was primarily designed for an image classification task rather than image-to-image mapping that we consider here.

## DiffNet: Discretisation and Implementation

In this section, we want to establish a layer operator based on the diffusion processes discussed in chapter 2. This means that we now interpret the layers $F_k$ of the continuum network as time states of the function $u: \varOmega \times \mathbb {R}_+ \rightarrow \mathbb {R}$, where *u* is a solution of the diffusion Eq. (2.1). In the following, we assume single-channel networks, i.e. $|F_k|=1$ for all layers. Then, we can associate each layer with the solution *u* such that $F_k=u^{(k)}=u(x,t=t_k)$. To build a network architecture based on the continuum setting, we introduce the layer operator versions of (2.10), and (2.20):

### Definition 7

(*Diffusion and filtering layer operator*) Given two layers $F_k$ and $F_{k-1}$, such that $F_k=u(x,t_k)$ and $F_{k-1}=u(x,t_{k-1})$, then a diffusion layer operator $\mathcal {D}_\varTheta$, with parameters $\varTheta =\{\gamma ,\delta t\}$, is given by

4.1 $$\begin{aligned} \begin{aligned} \mathcal {D}_\varTheta F_{k-1}&= \mathcal {D}_{\delta t}^\mathrm{Expl}(\gamma ^{(k-1)})u^{(k-1)} \\&= ({\hbox {Id}} + \delta t \mathcal {L}(\gamma ^{(k)})) u^{(k-1)} = F_k. \end{aligned} \end{aligned}$$

Similarly, an inverse filtering layer operator with parameters $\varTheta =\{\zeta ,\delta t \}$ is given by

4.2 $$\begin{aligned} \begin{aligned} \mathcal {E}_\varTheta F_{k-1}&= \mathcal {E}_{\delta t}(\zeta ^{(k-1)})u^{(k-1)} \\&=u^{(k-1)}- \delta t\int _{{\mathbb {R}}^d} \zeta (x,y) u^{(k-1)}(y) {\mathrm{d}}y = F_k. \end{aligned} \end{aligned}$$

Note that this formulation includes a learnable time step and hence the time instances that each layer represents changes. That also means that a stable step size is implicitly learned, if there are enough layers. In the following, we discuss a few options on the implementation of the above layer operator, depending on the type of diffusivity.

### Remark 4

The assumption of a single-channel network, i.e. $|F_k|=1$ for all *k*, can be relaxed easily, either by assuming $|F_k|=m$ for some $m \in \mathbb {N}$ and all layers, or by introducing a channel mixing as in the convolutional operator (3.1).

As a natural application, we could consider RGB or hyperspectral images as a multichannel input. In that case, the filters would become a tensor representing both intra- and inter-channel mixing but still modelled as a diffusion process—see, for example, [9].

### Discretisation of a Continuum Network

Let us briefly discuss some aspects on the discretisation of a continuum network; we first start with affine linear networks, such as the convolutional networks discussed in Sect. 3.2. Rather than discussing the computational implementation of a CNN, (see, for example, the comprehensive description in [10]), we concentrate instead on an algebraic matrix–vector formulation that serves our purposes.

For simplicity, we concentrate on the two-dimensional $d=2$ case here. Let us then assume that the functions $f_i$ in each layer are represented as a square *n*-by-*n* image and we denote the vectorised form as $\mathbf f \in \mathbb {R}^{n^2}$. Then, any linear operation on $\mathbf f$ can be represented by some matrix $\mathsf {A}$; in particular, we can represent convolutions as a matrix.

We now proceed by rewriting the convolutional operation (3.1) in matrix–vector notation. Given two layers $F_k$ and $F_{k-1}$ with channel index set $\mathbb {I},\ \mathbb {J}$, respectively, then we can represent the input layer as vector $\mathbf F _{k-1}\in \mathbb {R}^{Jn^2}$ and similarly the output layer $\mathbf F _{k}\in \mathbb {R}^{In^2}$. Let $\mathsf {A}_i\in \mathbb {R}^{n^2\times Jn^2 }$ represent the sum of convolutions in (3.1), then we can write the layer operator in the discrete setting as matrix–vector operation by

$$\begin{aligned} \mathbf f _i^k=\varphi (b_i\mathbb {1}+{\textsf {A}}_i \mathbf F _{k-1}), \end{aligned}$$

where $\mathbb {1}$ denotes a vector of ones with suitable size. Furthermore, following the above notation, we can introduce a stacked matrix $\mathsf {A}\in \mathbb {R}^{In^2\times Jn^2 }$ consisting of all $\mathsf {A}_i$ and a vector $\mathsf {b}\in \mathbb {R}^{I}$ consisting of all biases. Then, we can represent the whole convolutional layer in the discrete setting as

4.3 $$\begin{aligned} \mathbf F _k=\varphi (b\otimes \mathbb {1}+\textsf {A}{} \mathbf F _{k-1}). \end{aligned}$$

Now, the parameters of each layer are contained in the matrix $\mathsf {A}$ and vector $\mathsf {b}$.

### Learned Forward and Inverse Operators

**Fig. 1:**
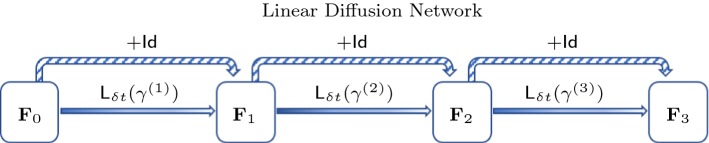
Illustration for a linear three-layer diffusion network. In this case, we learn the filters $\gamma$ as the diffusivity for each layer explicitly

**Fig. 2:**
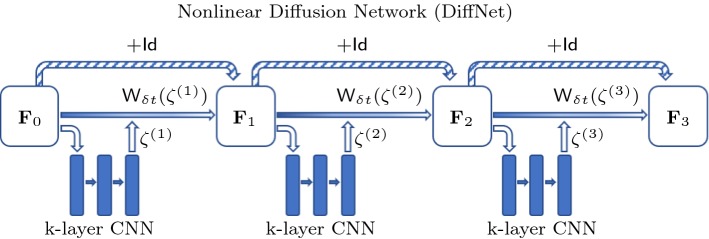
Illustration for a nonlinear three-layer diffusion network. Here, the filters $\zeta$ are implicitly estimated by a small *k*-layer CNN and then applied to the image in the filtering layer

Let us now discuss a similar construction for the diffusion layers. For the implementation of the diffusion network, we consider the explicit formulation in (2.10) with the differential operator $\mathcal {L}(\gamma ^{(k)}) = \nabla \cdot \gamma ^{(k)}\nabla$ approximated by the stencil (including the time step $\delta t$)

4.4 $$\begin{aligned} \mathsf {L}_{\delta t}(\gamma ^{(k)})= \delta t \begin{pmatrix} &{} \gamma _1^{(k)}&{}\\ \gamma _2^{(k)}&{}-\sum _i\gamma _i^{(k)} &{}\gamma _4^{(k)} \\ &{}\gamma _3^{(k)}&{} \end{pmatrix}. \end{aligned}$$

We use zero Neumann boundary conditions on the domain boundary

4.5 $$\begin{aligned} \partial _\nu u =\ 0 \text { on } \partial \varOmega \times (0,T]. \end{aligned}$$

Then, we can represent (4.1) by

4.6 $$\begin{aligned} \mathbf F _{k} = (\mathsf {Id}+ \mathsf {L}_{ \delta t}(\gamma )) \mathbf F _{k-1}. \end{aligned}$$

The basis of learning a diffusion network is now given as estimating the diagonals of $\mathsf {L}_{\delta t}(\gamma )$ and the time step $\delta t$. This can be done either explicitly as for the CNN or indirectly by an estimator network, as we will discuss next.

### Formulating DiffNet

Let us first note that if we construct our network by strictly following the update in (4.6), we restrict ourselves to the forward diffusion. To generalise the inverse problem, we consider

4.7 $$\begin{aligned} {\mathsf {W}}_{\delta t}(\zeta ) = \delta t \begin{pmatrix} &{}\zeta _1 &{}\\ \zeta _2&{} -\zeta _5 &{}\zeta _4 \\ &{} \zeta _3 &{} \end{pmatrix}. \end{aligned}$$

Additionally, there are two fundamentally different cases for the diffusivity $\gamma$ we need to consider before formulating a network architecture to capture the underlying behaviour. These two cases are

(i) Linear diffusion; spatially varying and possible time dependence, $\gamma =\gamma (x,t)$.
(ii)) Nonlinear diffusion; diffusivity depending on the solution *u*, $\gamma =\gamma (u(x,t))$.

In the first case, we could simply try to learn the diffusivity explicitly, to reproduce the diffusion process. In the second case, this is not possible, and hence, an estimation of the diffusivity needs to be performed separately in each time step from the image itself, before the diffusion step can be performed. This leads to two conceptually different network architectures.

The linear case (i) corresponds to the diffusion layer operator (4.1) and is aimed to reproduce a linear diffusion process with fixed diffusivity. Thus, learning the mean-free filter suffices to capture the physics. The resulting network architecture is outlined in Fig. 1. Here, the learned filters can be directly interpreted as the diffusivity of layer *k* and are then applied to $\mathbf F _{k-1}$ to produce $\mathbf F _k$.

In the nonlinear case (ii), we follow the same update structure, but now the filters are not learned explicitly; they are rather estimated from the input layer itself, as illustrated in Fig. 2. Furthermore, since this architecture is designed for inversion of the nonlinear diffusion process, we employ the generalised stencil ${\mathsf {W}}_{\delta t}(\zeta )$. Then, given layer $\mathbf F _k$, the filters $\zeta$ are estimated by a small CNN from $\mathbf F _{k-1}$, which are then applied following an explicit update as in (4.6) to produce $\mathbf F _k$. Note that the diagonals in the update matrix are produced by the estimation CNN. We will refer to this nonlinear filtering architecture as the *DiffNet* under consideration in the rest of this study.

In contrast to classical CNN architectures, the proposed DiffNet is nonlinear by design, and hence, no additional nonlinearities are necessary. Compared to previous approaches, such as [6], we note that the parameters of the estimating CNN are not used to process the image directly, but rather to produce the filters $\zeta$ for the update matrix only.

Comparing ’Diffnet’ to ’PDE-NET2.0’ [27], in the latter, the training assumes that full time-series data *u*(*x*, *t*) are available, and the PDE is approximated by a forward Euler method with appropriate stability constraints. In our approach, only initial and final conditions are assumed to be known; the coefficients of the PDE are spatially varying, and both a *forward* and an *inverse* problem can be learned, and the latter requires regularisation which is learned simultaneously with the PDE coefficients.

#### Implementation

The essential part for the implementation of a diffusion network is to perform the update (4.6) with either $\mathsf {L}_{\delta t}(\gamma )$ or $\mathsf {W}_{\delta t}(\zeta )$. For computational reasons, it is not practical to build the sparse diagonal matrix and evaluate (4.6); we rather represent the filters $\gamma$ and $\zeta$ as an $n\times n$-image and apply the filters as pointwise matrix–matrix multiplication to a shifted and cropped image, according to the position in the stencil. This way, the zero Neumann boundary condition (4.5) is also automatically incorporated.

For the linear diffusion network, we would need to learn the parameter set $\varTheta$, consisting of filters and time steps, explicitly. This has the advantage of learning a global operation on the image where all parameters are interpretable, but it comes with a few disadvantages. First of all, in this form, we are limited to linear diffusion processes and a fixed image size. Furthermore, the parameters grow with the image size, i.e. for an image of size $n\times n$, we need $5 n^2$ parameters per layer. Thus, applications may be limited.

**Fig. 3:**
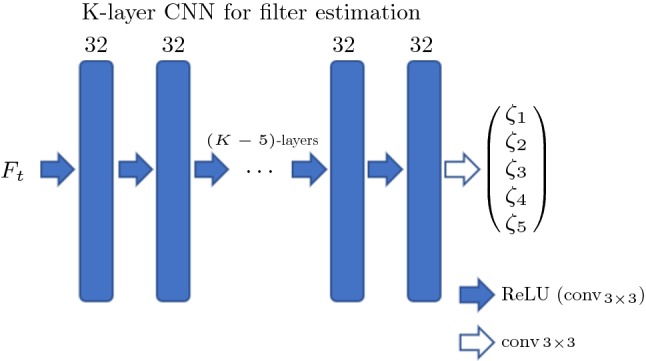
Architecture of the *K*-layer CNN used as diffusivity estimator in the nonlinear diffusion network (DiffNet)

For the nonlinear architecture of DiffNet, where the filters depend on the image at each time step, we introduced an additional estimator consisting of a *K*-layer CNN. This CNN gets an image, given as layer $\mathbf F _k$, as input and estimates the filters $\zeta$. The architecture for this *K*-layer CNN as estimator is chosen to be rather simplistic, as illustrated in Fig. 3. The input $\mathbf F _k$ consists of one channel, which is processed by $K-1$ convolutional layers with 32 channels and a ReLU nonlinearity, followed by the last layer without nonlinearity and five channels for each filter, which are represented as matrices of the same size as the input $\mathbf F _k$. In particular, for the chosen filter size of $3\times 3$, we have exactly $9\cdot (32+32\cdot 5 + 32^2\cdot (K-2))$ convolutional parameters and $32\cdot (K-1)+5$ biases per diffusion layer. That is for a five-layer CNN, we have 29.509 parameters independent of image size.

## Computational Experiments

In the following, we will examine the reconstruction capabilities of the proposed DiffNet. The experiments are divided into a simple case of deconvolution, where we can examine the learned features and a more challenging problem of recovering an image from its nonlinear diffused and noise-corrupted version.

### Deconvolution with DiffNet

We first examine a simple deconvolution experiment to determine what features the DiffNet learns in an inverse problem. For this task, we will only consider deconvolution without noise.

**Fig. 4:**
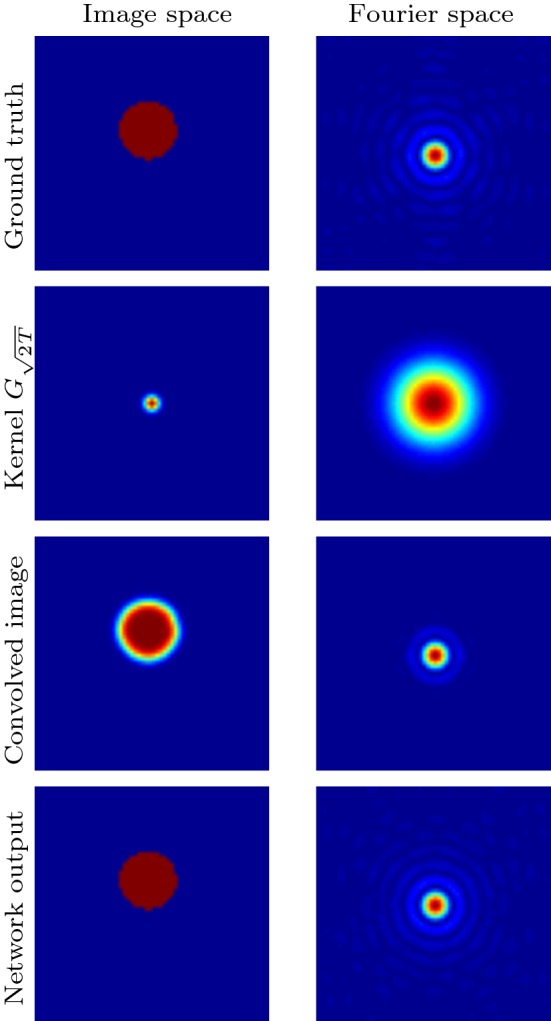
Illustration of the deconvolution problem for a simple ball. Left column shows the image space, and the right column shows the corresponding absolute value of the Fourier coefficients. All images are plotted on their own scale

**Fig. 5:**
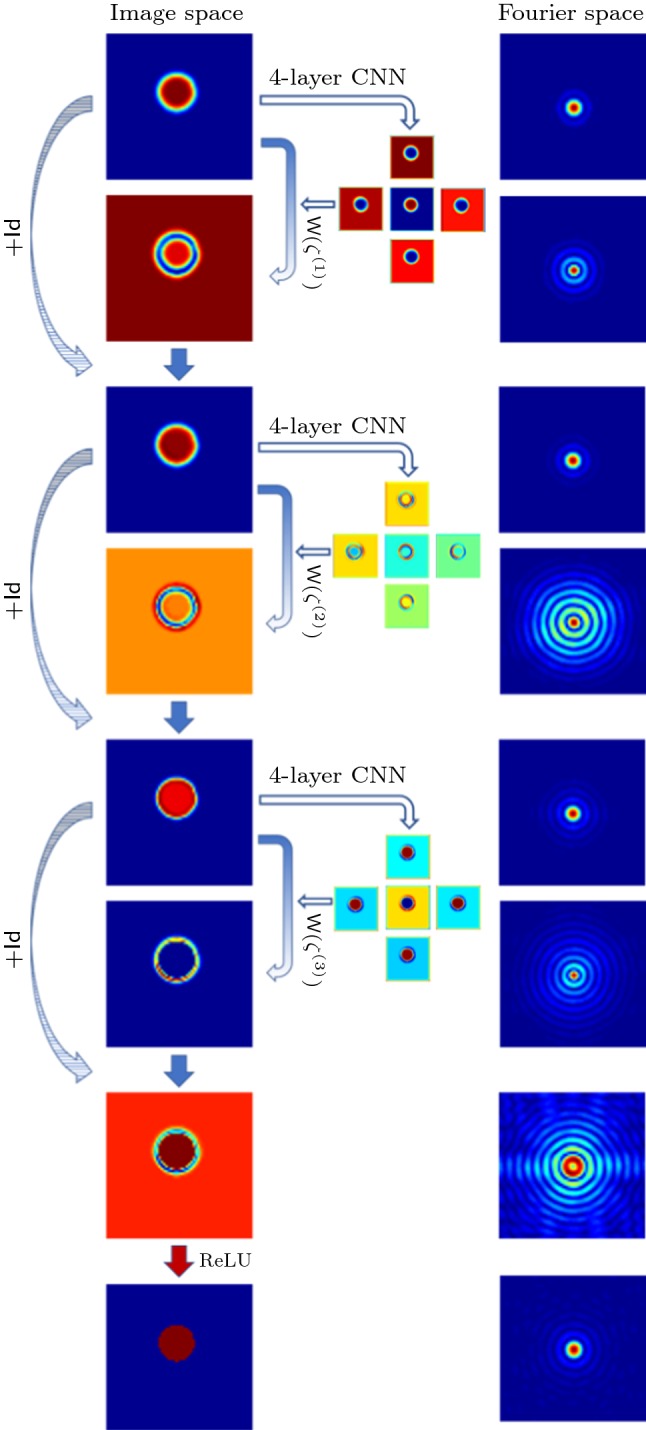
Illustration of the deconvolution process with three layers of DiffNet. The left column shows the processed image and intermediate steps. The right column shows the corresponding absolute value of Fourier coefficients. All images are plotted on their own scale

The forward problem is given by (2.1) with zero Neumann boundary condition (4.5) and constant diffusivity $\gamma \equiv 1$. For the experiment, we choose $T=1$, which results in a small uniform blurring, as shown in Fig. 4. We remind that for the isotropic diffusion, the forward model is equivalent to convolution in space with the kernel $G_{\sqrt{2T}}$, see (2.3). As it is also illustrated in Fig. 4, convolution in the spatial domain is equivalent to multiplication in Fourier domain. In particular, high frequencies get damped and the convolved image is dominated by low frequencies. Hence, for the reconstruction task without noise, we essentially need to recover the high frequencies.

The training and test data for DiffNet consist of simple discs of varying radius and contrast. The training set consists of 1024 samples and the test set of an additional 128, each of size $64\times 64$. The network architecture is chosen following the schematic in Fig. 2, with three diffusion layers and a final projection to the positive numbers by a ReLU layer. The filter estimator is given by a four-layer CNN, as described in Sect. 4.3.1. All networks were implemented in Python with TensorFlow [1].

The input to the network is given by the convolved image without noise, and we have minimised the $\ell ^2$-loss of the output to the ground-truth image. The optimisation is performed for about 1000 epochs in batches of 16 with the Adam algorithm and initial learning rate of $4\,\times \,10^{-4}$ and a gradual decrease to $10^{-6}$. Training on a single Nvidia Titan Xp GPU takes about 24 min. The final training and test error are both at a PSNR of 86.24, which corresponds to a relative $\ell ^2$-error of $2.5\,\times \,10^{-4}$. We remind that this experiment was performed without noise.

The result of the network and intermediate updates for one example from the test data are illustrated in Fig. 5. We also show the filters $\zeta ^{(k)}$ computed as the output of the trained CNN in each layer, $k = 1,2,3$. The output of the last diffusion layer $\mathbf F _3$ is additionally processed by a ReLU layer to enforce positivity in the final result. It can be seen that the network gradually reintroduces the high frequencies in the Fourier domain; especially, the last layer mainly reintroduces the high frequencies to the reconstruction. It is interesting to see that the learned filters follow indeed the convention that the central filter is of different signs than the directional filters. This enforces the assumption that the filter consists of a mean-free part and a regularising part, which should be small in this case, since we do not have any noise in the data. Lastly, we note that the final layer, before projection to the positive numbers, has a clearly negative part around the target, which will be cut off resulting in a sharp reconstruction of the ball.

**Fig. 6:**
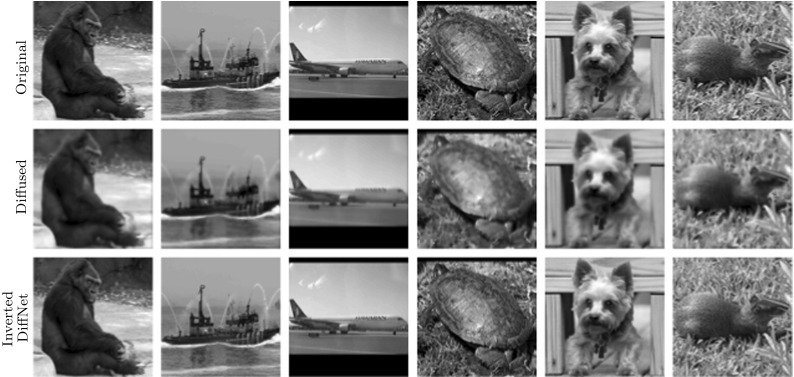
Samples from the test data for learning the inversion of nonlinear diffusion without noise. Mean PSNR for reconstructed test data with DiffNet is: 63.72

### Nonlinear Diffusion

Let us now consider the nonlinear diffusion process with the Perona–Malik filter function [30] for (2.1) with zero Neumann boundary condition (4.5). In this model, the diffusivity is given as a function of the gradient

5.1 $$\begin{aligned} \gamma (|\nabla u|^2) = \frac{1}{1+|\nabla u|^2/\lambda ^2} \end{aligned}$$

with contrast parameter $\lambda > 0$. We mainly concentrate here on the inverse problem of restoring an image that has been diffused with the Perona–Malik filter and contaminated by noise.

**Fig. 7:**
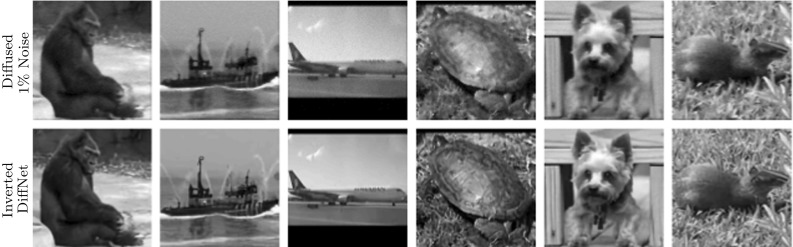
Samples from the test data for learning the inversion of nonlinear diffusion with $1\%$ noise. Mean PSNR for reconstructed test data with DiffNet is: 34.21

For the experiments, we have used the test data from the STL-10 database [7], which consists of 100,000 RGB images with resolution $96\times 96$. These images have been converted to grey scale and divided to 90,000 for training and 10,000 for testing. The obtained images were then diffused for four time steps with $\delta t = 0.1$ and $\lambda =0.2$. A few sample images from the test data with the result of the diffusion are displayed in Fig. 6. The task is then to revert the diffusion process with additional regularisation to deal with noise in the data.

For all experiments, we have used the same network architecture of DiffNet using the architecture as illustrated in Fig. 2. By performing initial tests on the inversion without noise, we have found that five diffusion layers with a four-layer CNN, following the architecture in 3, gave the best trade-off between reconstruction quality and network size. Increasing the amount of either layers led to minimal increase in performance. Additionally, we have used a ReLU layer at the end to enforce nonnegativity of the output, similarly to the last experiment. We emphasise that this architecture was used for all experiments and hence some improvements for the high-noise cases might be expected with more layers. All networks were trained for 18 epochs, with a batch size of 16, and $\ell ^2$-loss. For the optimisation, we have used the Adam algorithm with initial learning rate of $2\,\times \,10^{-3}$ and a gradual decrease to $4\,\times \,10^{-6}$. Training on a single Nvidia Titan Xp GPU takes about 75 min.

As benchmark, we have performed the same experiments with a widely used network architecture known as U-Net [32]. This architecture has been widely applied in inverse problems [3, 12, 21, 22], even for applications where it is theoretically unsuitable, and hence can be considered as an established benchmark. It is mainly used to post-process initial directly reconstructed images from undersampled or noisy data, for instance, by filtered back-projection in X-ray tomography or the inverse Fourier transform in magnetic resonance imaging [16]. The network architecture we are using follows the publication [21] and differs from the original mainly by a residual connection at the end. That means the network is trained to remove noise and undersampling artefacts from the initial reconstruction. In our context, the network needs to learn how to remove noise and reintroduce edges. For training, we have followed a similar protocol as for DiffNet. The only difference is that we started with an initial learning rate of $5\,\times \,10^{-4}$ with a gradual decrease to $2\,\times \,10^{-5}$. Training of U-Net takes about 3 h.

**Fig. 8:**
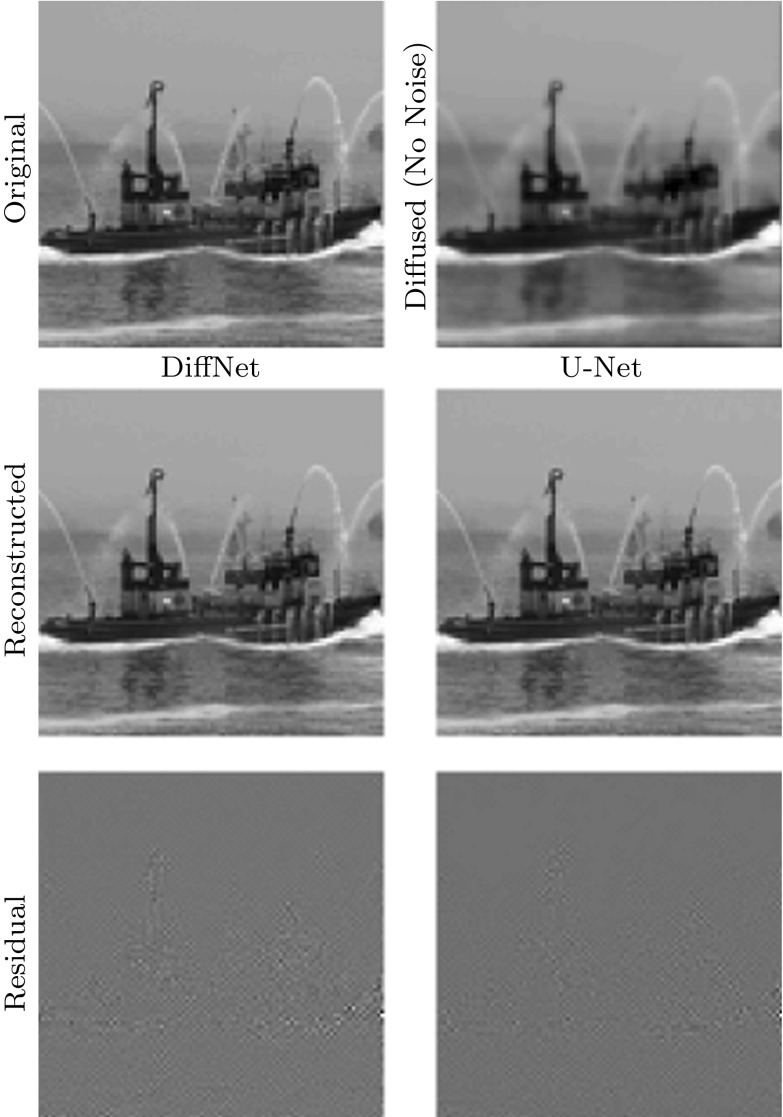
Comparison of reconstruction quality for reconstruction from nonlinear diffused image without noise. Both networks are trained on the full set of 90,000 images. PNSR: DiffNet 65.34, U-Net 61.08

**Fig. 9:**
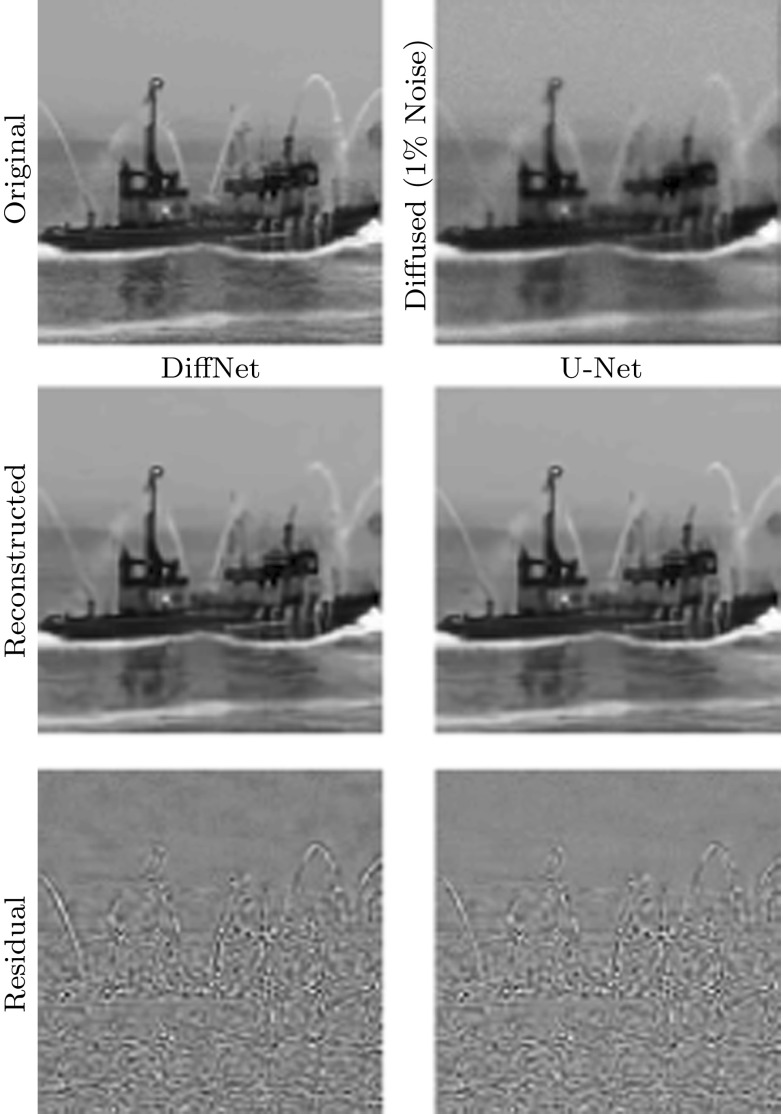
Comparison of reconstruction quality for reconstruction from 1% noise contaminated nonlinear diffused image. Both networks are trained on the full set of 90,000 images. PNSR: DiffNet 34.96, U-Net 35.27

The reconstruction results, for some samples of the test data, with DiffNet can be seen in Fig. 6 for the case without noise and in Fig. 7 for 1% noise on the diffused images. A comparison of the reconstruction results with U-Net and DiffNet is shown in Fig. 8 for the test without noise and in Fig. 9 for 1% noise. Qualitatively, the reconstructed images are very similar, as can be seen in the residual images in the last column. The leftover noise pattern for both networks is concentrated on the fine structures of the ship. Quantitatively, for the noise-free experiment, DiffNet has an increase of 4 dB in PSNR compared to the result of U-Net, 65.34 (DiffNet) compared to 61.08 (U-Net). For the case with 1% noise, the quantitative measures are very similar. Here, U-Net has a slightly higher PSNR with 35.27 compared to DiffNet with 34.96. A thorough study of reconstruction quality of both networks follows in the next section as well as some interpretation of the learned features in DiffNet.

**Fig. 10:**
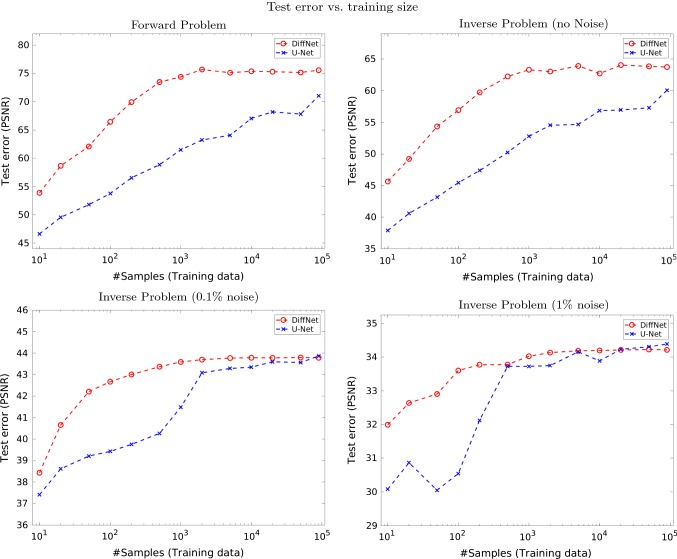
Generalisation plot for the forward and inverse problems of nonlinear diffusion and varying noise levels. Test error depending on the amount of training data, for both DiffNet and U-Net

## Discussion

First of all, we note that the updates in DiffNet are performed explicitly and that the CNN in the architecture is only used to produce the filters $\zeta$. This means that DiffNet needs to learn a problem-specific processing, in contrast to a purely data-driven processing in a CNN. Consequently, the amount of necessary learnable parameters is much lower. For instance, the five-layer DiffNet used for inversion of the nonlinear diffusion in Sect. 5.2 has 101,310 parameters, whereas the used U-Net with a filter size of $3\times 3$ has a total of 34,512,705 parameters, i.e. DiffNet uses only $\sim 0.3\%$ of parameters compared to U-Net, and hence, the learned features can be seen to be much more explicit. In the following, we discuss some aspects that arise from this observation, such as generalisability and interpretability.

**Fig. 11:**
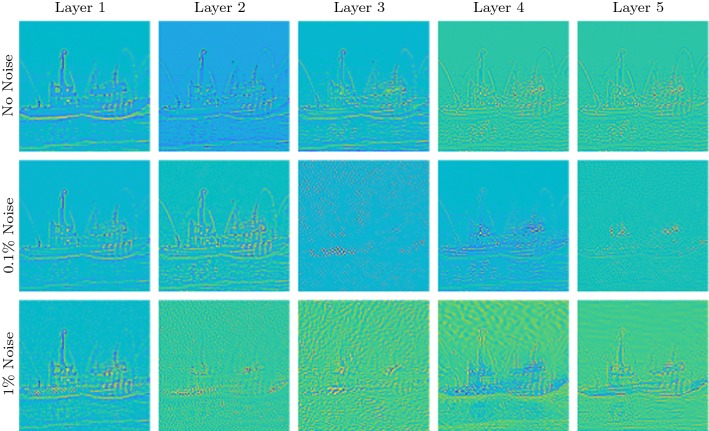
Filter updates $\sum _{i=1}^4 \zeta _i - \zeta _5$ for different noise levels. Each image is displayed on its own scale

**Fig. 12:**
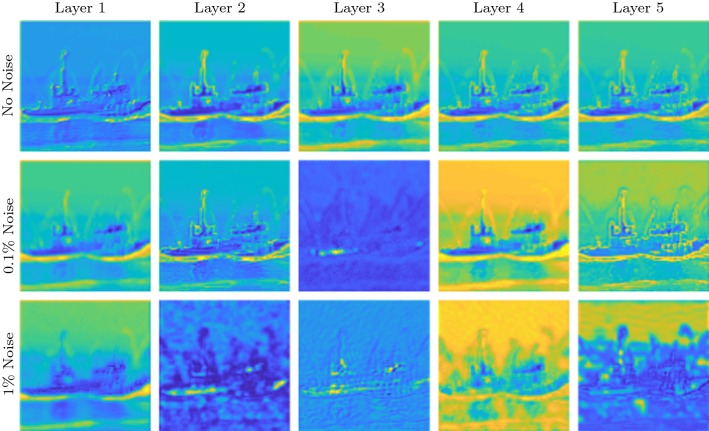
Obtained diagonal filters $\zeta _5$ for different noise levels. Each filter is displayed on its own scale

### Generalisability

To test the generalisation properties of the proposed DiffNet, we have performed similar experiments as shown in Sect. 5.2 for nonlinear diffusion, but with increasing amounts of training data. Under the assumption that DiffNet learns a more explicit update than a classic CNN, we would expect also to require less training data to achieve a good test error. To certify this assumption, we have examined four settings of nonlinear diffusion with the Perona–Malik filter: learning the forward model, learning to reconstruct from the diffused image without noise, as well as with 0.1% and 1% noise. We then created training datasets of increasing size from just 10 samples up to the full size of 90,000. For all scenarios, we have trained DiffNet and U-Net following the training protocol described in 5.2. Additionally, we have aborted the procedure when the networks started to clearly overfit the training data.

Results for the four scenarios are shown in Fig. 10. Most notably DiffNet outperforms U-Net clearly for the forward problem and the noise-free inversion, by 4dB and 3dB, respectively. For the noisy cases, both networks perform very similar for the full training data size of 90,000. The biggest difference overall is that DiffNet achieves its maximum test error already with 500–1000 samples independent of the noise case, whereas the U-Net test error saturates earlier with higher noise. In conclusion, we can say that for the noisy cases, both networks are very comparable in reconstruction quality, but for small amounts of data, the explicit nature of DiffNet is clearly superior.

### Interpretation of Learned Filters

Since all updates are performed explicitly with the output from the filter estimation CNN, we can interpret some of the learned features. For this purpose, we show the filters for the ship image from Sect. 5.2 for the three inversion scenarios under consideration. In Fig. 11, we show the sum of all learned filters, i.e. $\sum _{i=1}^4 \zeta _i - \zeta _5$. If the network would only learn the mean-free differentiating part, then these images should be zero. This implies that the illustrated filters in Fig. 11 can be related to the learned regularisation $\mathsf {S}(\zeta )$. Additionally, we also show the diagonal filters $\zeta _5$ in Fig. 12.

We would expect that with increasing noise level, the filters will incorporate more smoothing to deal with the noise; this implies that the edges get wider with increasing noise level. This can be nicely observed for the diagonal filters in Fig. 12. For the smoothing in Fig. 11, we see that the first layer consists of broader details and edges that are refined in the noise-free case for increasing layers. In the noisy case, the latter layers include some smooth features that might depict the regularisation necessary in the inversion procedure. It is generally interesting to observe that the final layer shows very fine local details, necessary to restore fine details for the final output.

**Fig. 13:**
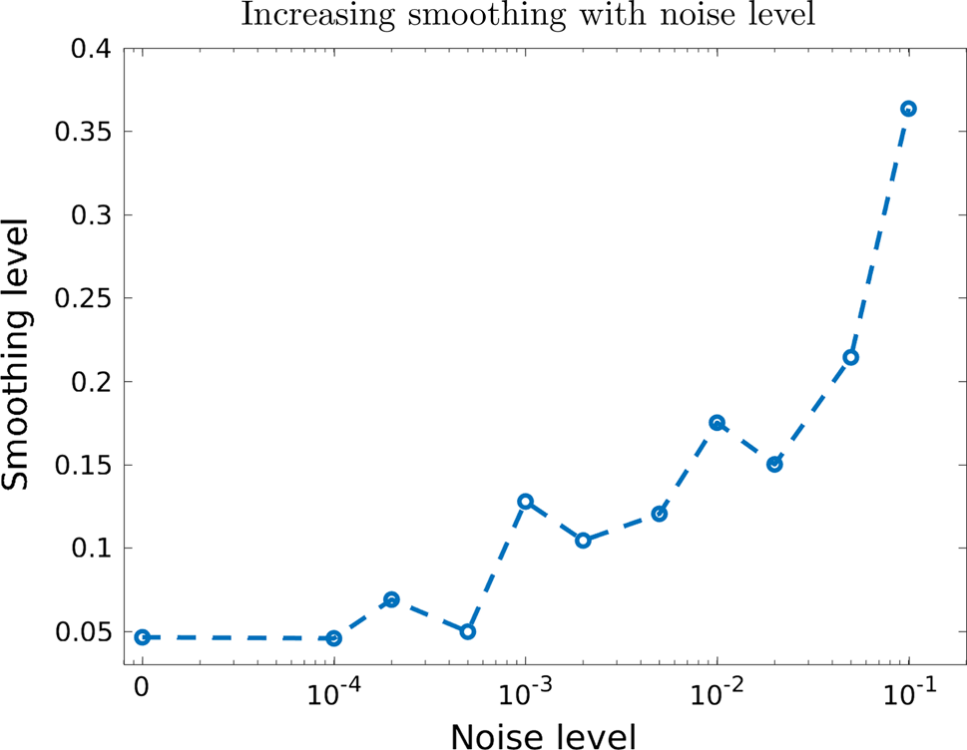
Estimate of the smoothing level $\alpha$ for increasing noise in the inverse problem. Computed over a sample of 32 images from the test data

Finally, we have computed training data of a wider noise range to examine the regularisation properties of the learned network. For this, we have taken the full 90,000 training samples and contaminated the diffused image with noise ranging from 0.01 to 10% noise. As we conjectured in Sect. 2.3, the learned update filters can be decomposed to a mean-free operation and a smoothing part $\mathsf {W}(\zeta )=\mathsf {L}(\zeta )+\mathsf {S}(\zeta )$. This implies that the magnitude of $\mathsf {S}(\zeta )$ has to increase with higher noise. To examine this conjecture, we have taken (fixed) 32 samples from the reconstructed test data for each noise level and computed an estimate of $\mathsf {S}$ as the sum $\sum _{i=1}^4 \zeta _i - \zeta _5$, i.e. the deviation from the mean-free part. Furthermore, we use the mean of the absolute value of $\mathsf {S}$ over the whole image as an estimator of $\alpha$. The resulting graph of smoothing versus noise level is shown in Fig. 13. As we have conjectured, the estimate of $\alpha$ increases clearly with the noise level, and hence, we believe our interpretation of the learned filters as the composition of a mean-free part and a smoothing necessary for ill-posed inverse problems is valid.

## Conclusions

In this paper, we have explored the possibility to establish novel network architectures based on physical models other than convolutions; in particular, we concentrated here on diffusion processes. As main contributions, we have introduced some nonlinear forward mappings, modelled through learning rather than just through PDEs or integral transforms. We have reviewed (regularised) inverse diffusion processes for inverting such maps. In particular, we have conjectured that these inverse diffusion processes can be represented by local non-stationary filters, which can be learned in a network architecture. More specific, these local filters can be represented by a sparse sub-diagonal (SSD) matrix and hence efficiently used in the discrete setting of a neural network. We emphasise that even though we have concentrated this study on a specific structure for these SSD matrices based on diffusion, other (higher order) models can be considered.

We obtain higher interpretability of the network architecture, since the image processing is explicitly performed by the application of the SSD matrices. Consequently, this means that only a fraction of parameters is needed in comparison with classical CNN architectures to obtain similar reconstruction results. We believe that the presented framework and the proposed network architectures can be useful for learning physical models in the context of imaging and inverse problems, especially where a physical interpretation of the learned features is crucial to establish confidence in the imaging task.

## Footnotes

1 Note the definition of Fourier Transform is chosen to give the correct normalisation so that $\hat{u}(k)|_{k=0} = \int _{{\mathrm {R}}^d} u(x) {\mathrm{d}}^n x.$
